# A Case for Case Report

**Published:** 2010-12-01

**Authors:** Vivek Gharpure

**Affiliations:** Children’s Surgical Hospital,13, Pushpanagari, Aurangabad, 431001, India

Evidence based medicine (EBM) is becoming popular among clinicians and medical publishers; as clinical research is tested against the touchstone of EBM. Theory of Quality of Evidence, considers randomized controlled trials to be the best quality evidence, while case reports and expert opinions are considered at the lowest ebb.

Most of us are not good at mathematics. We tend to forget that ‘evidence’ is not the ‘proof’. Evidence is information in support of a hypothesis. Scientific theories and rules have exceptions, considered as special cases. A theory that explains special cases is a better theory. Einstein’s general theory of relativity was verified on the basis of observations and it clarified special cases of Newton’s theory of gravity. Hard science works with hard facts. Hard facts prove hypotheses right or wrong. Scientific theories of biology and medicine are based on soft facts, thus need soft evidences. Observer bias enters in physics experiments too. It enters even more in biological observations. It can be fallacious to depend too much upon evidence in soft sciences.

Even in of mathematics, where everything is cut and dried and all theorems can be ‘proved’, Gödel’s incompleteness theorem took the ground away from mathematician’s feet, by stating that ‘there exist statements in mathematics which are true but cannot be proved using mathematical methods’. Compared to mathematics, evidence in medicine is as solid as a marshmallow. Edward Jenner was ridiculed by his physician colleagues for believing that cow pox prevented small pox; as in their opinion, the ‘evidence’ was inconclusive and unsatisfactory. Semmelweis was evicted from Vienna General Hospital for suggesting hand washing to prevent puerperal sepsis; which hypothesis was contrary to the contemporary evidence in favor of miasmas.

Great scientific discoveries like the Archimedes principle, structure of benzene, penicillin, x-rays, microwave radiation, to name a few, have been made by accident. These gentlemen had been working on a related problem while they serendipitously hit the jackpot. Had they not been perceptive enough, it might have taken many more years before someone else discovered it. ‘Chance favors the prepared mind’ Pasteur said. Few scientific discoveries are made by the voluntary technique of pure thought and reason. Scientific establishments take great pains to hide the facts. If an inspired guess turns to be correct, it is not reported as an inspired guess but a solid reasoning is thought up, after the fact, to give weight to it. Scientists have become used to being ashamed of inspired guesses, revelations, and insights [1]. 

Research is driven by hunches. A scientist does not start randomly but has a hunch about what he is likely to find. Hunches can be based on first principles, reading other scientists’ work, informal discussion with others etc. It is no fun trying to repeat what others have done and solve problems that have been solved before. A true scientist loves the challenge of sailing in uncharted waters. But like Columbus, he has to have a hunch. Developing good hunches is a matter of inclination, luck and inspiration [2]. Inspiration is what one likely to get from case reports. In medicine, more than any other science, we have to extrapolate seemingly unrelated information to come up with a solution.

Pediatric surgery deals with rare and uncommon conditions. We are fortunate to live in that part of the world where children form more than 30% of the population. Congenital defects are a form of statistical error. We see more patients of a particular condition in a month that many surgeons in developed world would see in a year. We see more unusual presentations of conditions than others. Editorial policies of established journals discourage case reports [3]. It is difficult for a single surgeon to accumulate a series of patients with an uncommon presentation of a condition which by itself is rare. Unless these are reported, others will never learn about them and might keep reinventing the wheel.

Case reports, increase the breadth of clinical knowledge; make more ‘bandwidth’ available in the brain. Confronted with a seemingly difficult problem, a solution can emerge. Case reports make more tools available to the surgeons. Reading case reports is like walking in a rain forest; one doesn’t know which beetle, one will find next. It is a curious thrill to browse through case reports. Like reading the agony column! A case report is like finding a differently colored shell or odd shaped pebble on the beach. It is about being perceptive, being enthusiastic. Case reports are not circus freak shows. They are important medical tools. Ambrose Pare managed but one patient initially with his method that revolutionized wound care [4]. HIV/AIDS was published as a case report in 1981 [5]. A perceptive physician is what is needed, to make useful discoveries in medicine.

Case reports give us inspirations. Who knows, one of the physicians might discover new syndrome one day? Case reports encourage original thinking and creativity. Let us hope creativity does not become a vestigial organ.

## Footnotes

**Source of Support:** Nil

**Conflict of Interest:** None declared

## References

[1] ( 1971). Asimov I. The Eureka Phenomenon. The Magazine of Fantasy and Science Fiction.

[2] (2010). Johnson S. Where good ideas come from: The natural history of innovation.

[3] ( 1985). Morgan PP. Why case reports?. Can Med Assoc J.

[4] (1960). Bishop WJ. The early history of Surgery.

[5] ( 1981). Gottlieb GJ, Ragaz A, Vogel JV, Friedman-Kien A, Rywlin AM, Weiner EA, et al. A preliminary communication on extensively disseminated Kaposi's sarcoma in young homosexual men. Am J Dermatopathol.

